# Alveolar soft-part sarcoma responding to interferon alpha-2b

**DOI:** 10.1038/sj.bjc.6601074

**Published:** 2003-07-15

**Authors:** K J Roozendaal, B de Valk, J J A ten Velden, H J van der Woude, B B R Kroon

**Affiliations:** 1 Department of Haematology/Medical Oncology, Onze Lieve Vrouwe Gasthuis, Eerste Oosterparkstraat 179, 1090 HM Amsterdam, The Netherlands; 2 Department of Pathology, Onze Lieve Vrouwe Gasthuis, Eerste Oosterparkstraat 179, 1090 HM Amsterdam, The Netherlands; 3 Department of Radiology, Onze Lieve Vrouwe Gasthuis, Eerste Oosterparkstraat 179, 1090 HM Amsterdam, The Netherlands; 4 Department of Surgery, The Netherlands Cancer Institute/Antoni van Leeuwenhoek Hospital, Plesmanlaan 121, 1066 CX Amsterdam, The Netherlands

**Keywords:** alveolar soft-part sarcoma, interferon alpha-2b, antiangiogenic activity

## Abstract

A 23-year-old woman with an alveolar soft-part sarcoma of her calf with pulmonary metastases unresponsive to chemotherapy is described. Interferon (IFN) alpha-2b induced an impressive tumour response still ongoing after IFN treatment had to be stopped because of a psychosis. An explanation of this effect is still speculative.

Alveolar soft-part sarcoma (ASPS), a rare soft tissue sarcoma in young adults, preferentially presents as a quite large, painless tumour mass in the lower extremities. The microscopic picture shows an organoid nest-like arrangement of large polygonal cells, surrounded by thin-walled vascular channels. Mitotic figures are scarce. Central degeneration with loss of cohesion in the nests often provides a pseudoalveolar pattern. The cells contain varying amounts of glycogen and ‘diagnostic’ PAS-positive diastase-resistant crystalline material. Frequently, numerous multiple arteriovenous shunts are seen in the tumour (Enzinger and Weiss, 2001), across which sometimes a murmur can be heard (van Ruth *et al*, 2002). The histogenesis is still unresolved. A skeletal muscle origin of the tumour cells has been assumed, but recent studies with application of more advanced techniques did not confirm this concept (Gomez *et al*, 1999). Cytogenetic analysis revealed a clonal translocation between chromosomes X and 17 (Joyama *et al*, 1999). Unresectable disease or metastases at onset are present in about 35–50%, but usually metastases to lung, brains and bone appear later. Alveolar soft-part sarcoma generally progresses slowly, with a median survival of 48 months and an overall 5-year survival of 38% in young adults. The ultimate prognosis is still poor. There is only a partial response on chemotherapy in various combinations, in about 30% (Casanova *et al*, 2000; van Ruth *et al*, 2002).

We describe a 24-year-old woman with chemoresistant ASPS showing a still ongoing good partial response to interferon (IFN) alpha-2b.

## CASE REPORT

A 24-year-old woman presented in June 2000 with a fast-growing, tender swelling of her right calf without systemic complaints. Magnetic resonance imaging (MRI) showed a tumour of the gastrocnemius muscle around the right fibula of 5.5 × 8 cm without invasion of surrounding structures. Incision biopsy of the tumour (July 2000) revealed the characteristic histological pattern of ASPS with diastase-resistant PAS-positive accumulations of crystalline material identified in the cytoplasm. The cells showed immunoreactivity for vimentin, desmin and for S-100 protein. Other muscle markers (SMA, HHF-35), melanoma markers (HMB-45, KBA-62), and epithelial markers (Lu-5, CAM 5.2) were negative. Isolated limb perfusion was considered, but the thorax computed tomography (CT) scan revealed multiple, pulmonary metastases on both sides that were not resectable. Computed tomography scans of abdomen and brain were normal.

## RESULTS OF TREATMENT

**Figure 1:**
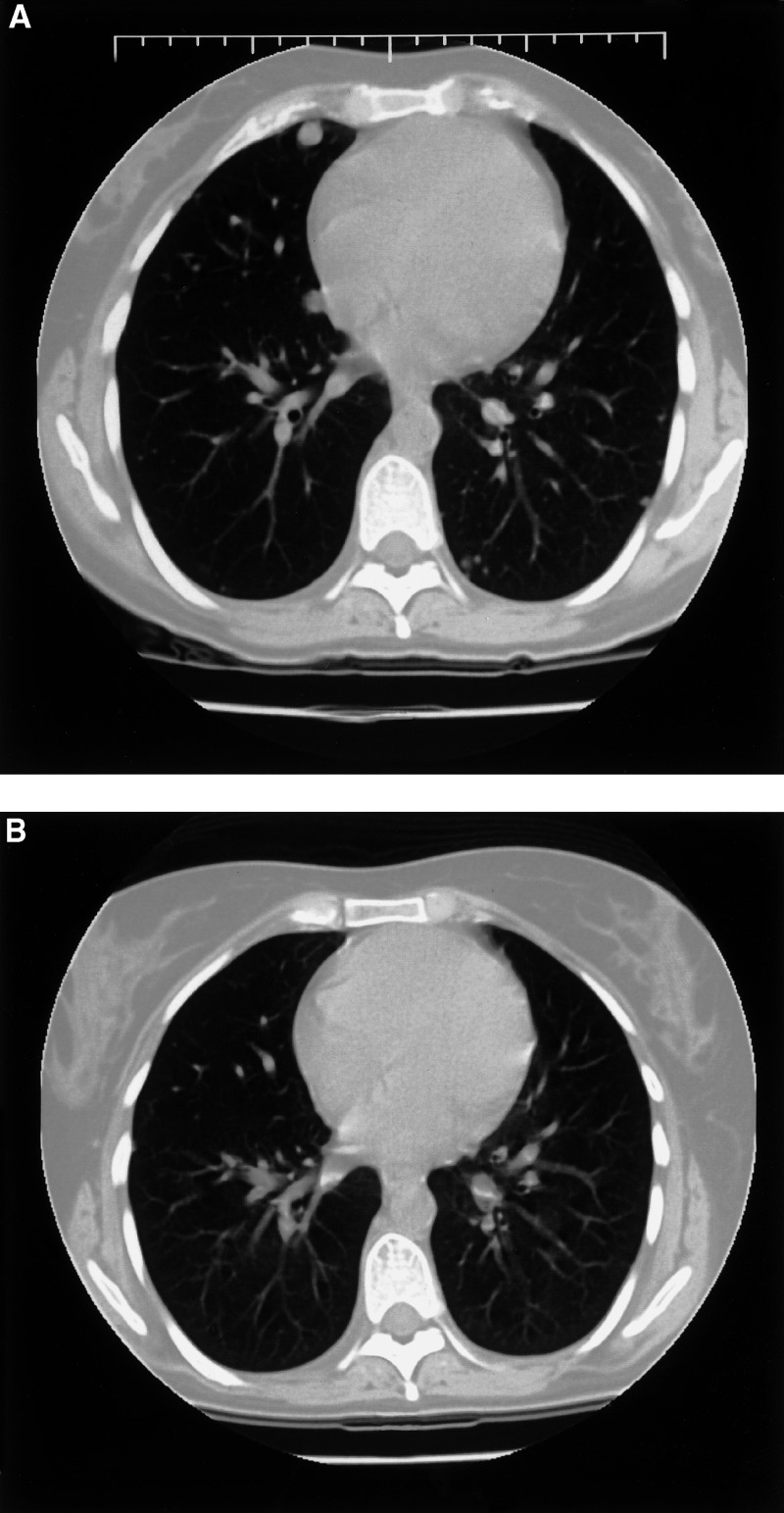
Axial CT section after three courses of chemotherapy (**A**) reveals multiple nodules of variable size consistent with metastases with a predominantly subpleural localisation. After IFN alpha-2b therapy, almost complete disappearance of the multiple subpleural metastases is shown (**B**).

**Figure 2:**
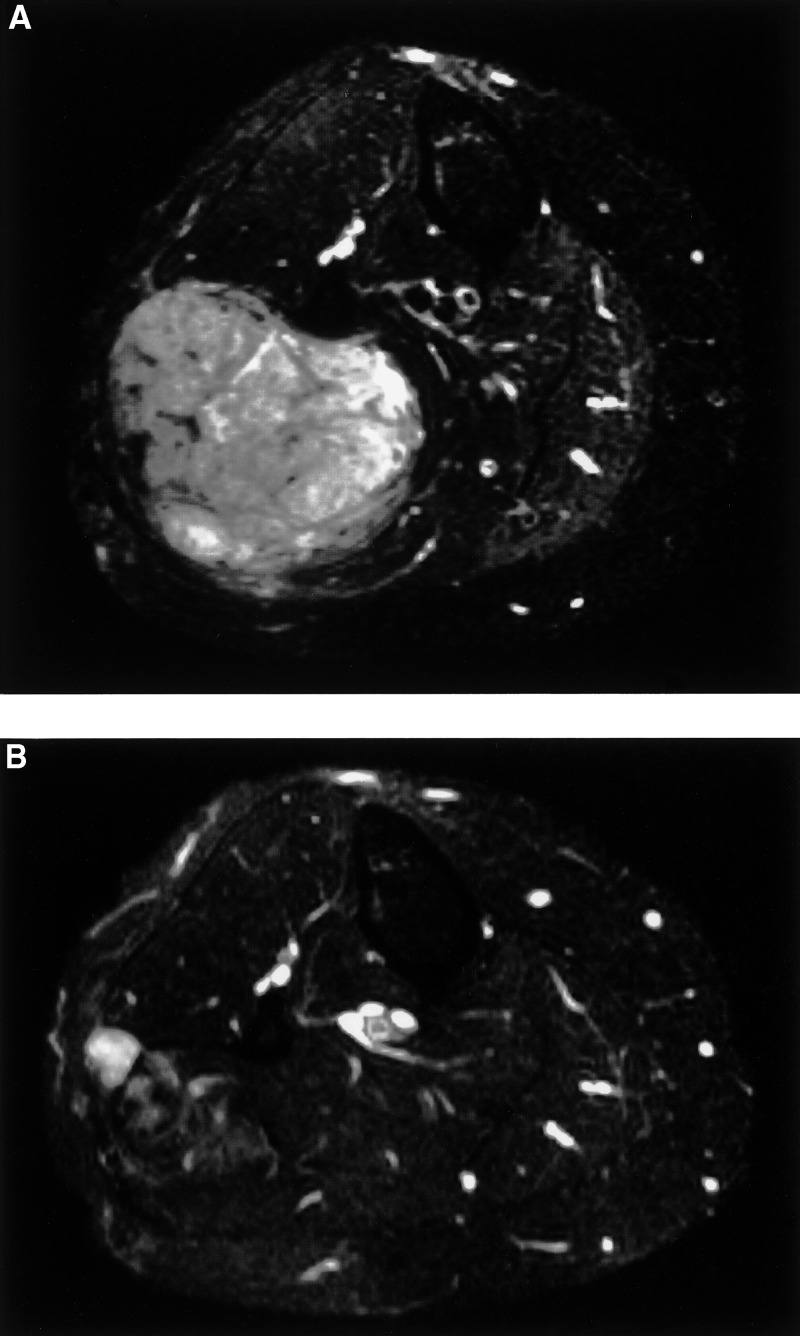
Fat-suppressed T2-weighted axial MR image after three courses of chemotherapy (**A**). A relatively well-defined mass of heterogeneous high signal intensity is shown in the right calf. Compared with the prechemotherapy MR examination (not shown), a small increase in tumour volume was noticed. After treatment with IFN alpha-2b, there is an obvious decrease of the tumour in the right calf with an overall decrease in signal intensity and a focal remnant area of high signal intensity on the lateral side (**B**).

After treatment with three courses of intensive chemotherapy according to the SIOP-MTT-98 study for metastatic malignant soft tissue sarcomas of childhood, the lung metastases (Figure 1A

) and primary tumour (Figure 2A

) showed obvious progression. High-dose chemotherapy followed by peripheral stem cell transplantation was considered too toxic. After three oral etoposide and three topotecan courses, progression continued. In April 2001, treatment with IFN alpha-2b (3 × 10^6^ IU s.c. day^−1^), was started, resulting in a gradual softening of the primary tumour. After 3 months, the dose was increased to 5 × 10^6^ IU s.c.day^−1^, and after a further 3 months, a good partial regression of the pulmonary metastases (Figure 1B) and tumour in the calf (Figure 2B) was confirmed. Two months later (December 2001), IFN alpha-2b had to be discontinued because the patient developed an acute psychotic syndrome. Contrast-enhanced CT scan of the brain revealed no abnormalities. After stopping IFN alpha-2b, she still remains in a good partial remission and has almost completely recovered from her psychosis.

## DISCUSSION

Primary conservative surgery is the mainstay of therapy of ASPS, eventually preceded by isolated limb perfusion in case of questionable resectability or followed by local radiation. The ASPS of the patient in this case report was clearly progressive during a 4 months lasting treatment with three combination chemotherapy schemes and two periods of single-agent treatment. In an ultimate effort to inhibit the progressive growth of this well-vascularised tumour, treatment with the relatively weak and nonspecific angiogenesis inhibitor IFN alpha-2b was initiated. After a 6 months lasting period of stable disease, this treatment resulted in an impressive partial regression of primary tumour and lung metastases. Although treatment had to be stopped because of an acute psychotic syndrome, interpreted as side effects of the interferon, the remission now lasts for 14 months, while the patient has recovered from her psychosis. A comparable observation has recently been described in an 8-year-old boy (Kuriyama *et al*, 2001).

The explanation for regression of ASPS after IFN alpha treatment is still speculative. Growth inhibition may be mediated by IFN alpha-receptors shown to be present in most solid tumours in childhood (Rosolen *et al*, 1997). However, information about expression of this receptor in ASPS is lacking. Otherwise, the efficacy of IFN alpha may be attributed to growth inhibition of microvascular endothelial cells as described in haemangiomas of infancy, by inhibiting basic fibroblast growth factor (bFGF) overproduction by tumour cells (Folkman, 1989) or by modulation of the immune effector cells.
